# A Novel Strategy for Single-Session Ultrasound-Guided Radiofrequency Ablation of Large Benign Thyroid Nodules: A Pilot Cohort Study

**DOI:** 10.3389/fendo.2020.560508

**Published:** 2020-10-07

**Authors:** Zhicheng Yao, Tao Wu, Bowen Zheng, Lei Tan, Yufan Lian, Bo Liu, Jie Ren

**Affiliations:** ^1^Department of General Surgery, Third Affiliated Hospital of Sun Yat-sen University, Guangzhou, China; ^2^Department of Interventional Ultrasound, Third Affiliated Hospital of Sun Yat-sen University, Guangzhou, China

**Keywords:** ultrasound-guided ablation, radiofrequency ablation, hydrodissection, benign thyroid nodule, CEUS (contrast-enhanced ultrasound)

## Abstract

**Background:** Ultrasound-guided radiofrequency ablation (RFA) of thyroid nodules (TNs) is a minimally invasive procedure that has been widely used to induce volume reduction in symptomatic solid benign TNs. The goal of this study was to investigate a novel therapeutic approach for single-session ablation of large thyroid nodules (LTNs, vol > 20 ml).

**Methods:** We performed a pilot cohort study of 21 patients with symptomatic solid benign LTNs (vol > 20 ml), who accepted ultrasound-guided RFA treatment between September 2018 and November 2019. RFA was performed using an 18-gauge internally cooled electrode with ultrasonographic guidance in a single session combined with intraoperative hydrodissection and immediate contrast-enhanced ultrasound (CEUS) to optimize safety and efficacy. Nodule volume was evaluated before ablation and at 1, 3, and 6 months after initial ablation, and all patients were asked to assess the cosmetic score (from 1 to 4) and symptom score (from 0 to 10) before ablation and at every follow-up after ablation.

**Results:** At the 6 month follow-up, there was significant nodule volume reduction, from 27.49 ml ± 7.9 (standard deviation) to 3.82 ml ± 5.02 (*p* = 0.001). Cosmetic signs (*p* = 0.001) and pressure symptoms (*p* = 0.001) were significantly improved. All patients underwent RFA without any major complications, and very few patients developed a change in voice (2/21). However, the changes subsided within 1 month. Almost half of the patients received an additional RFA (11/21) treatment to achieve complete ablation on the intraoperative immediate CEUS evaluation.

**Conclusion:** RFA is effective for treating LTNs (vol > 20 ml) and controlling clinical symptoms with a low complication rate. Patients were satisfied with cosmetic sign and pressure symptom improvement. The intraoperative hydrodissection and immediate CEUS represent a novel therapeutic approach for single-session ablation of LTNs.

## Introduction

Recently, thyroid nodules (TNs) have become a more common clinical problem, with a high incidence resulting from increased use of thyroid ultrasonography (US) (1). Epidemiologic studies have shown that palpable prevalence of TNs is very high in the iodine-sufficient parts of the world, especially for women and the elderly (2, 3). Most TNs are benign, but the vast majority of palpable TNs require treatment for the compressive symptoms and for cosmetic reasons (4, 5).

Thyroid surgery is the main treatment for symptomatic TNs, but it has several drawbacks (6). Radiofrequency ablation (RFA) is a non-surgical, minimally invasive procedure that was introduced for TN treatment in 2006 and has been proven to be an effective and safe procedure for treating benign TNs by many recent studies (7, 8). Consequently, RFA has also been recommended by several mainstream guidelines, such as those of the American Association of Clinical Endocrinologists, the American College of Endocrinology, Associazione Medici Endocrinologi (AACE/ACE/AME), and the Korean Society of Thyroid Radiology (KSTR) (9, 10).

As TNs are a benign disease, the major goals of treatment are to effectively reduce TN volume, as well as to relieve the compressive symptoms, cosmetic problems, and related anxiety. According to the current literature, RFA for benign TNs can reach this goal effectively, with a volume reduction rate (VRR) from 33 to 58% after 1 month and 51 to 85% after 6 months, indicating its ability to resolve most nodule-related clinical symptoms (8, 11–14).

BTNs with a large initial volume (vol > 20 ml) rarely achieve complete ablation during only one RFA treatment session. The large nodule occupies vast space, and if situated close to vital structures, ablation difficulty is increased, even for skillful doctors. US-guided ablation for large nodules is most likely to leave some unablated residual, which can result in failure to resolve clinical symptoms (15). Then, additional ablation sessions must be performed, resulting in increased medical costs and pain to subjects. A prospective randomized controlled study has proven that single-session RFA could achieve a satisfactory clinical response in most patients, but not for those patients with a large nodule (vol > 20 ml) (16). A 4-year follow-up study showed that the complete ablation of BTNs is important for preventing regrowth after RFA (17). All recurrent nodules come from the untreated residual left by the previous RFA (18).

Therefore, a new strategy of single-session treatment for large benign TNs is needed to ensure maximal ablation without impacting safety. Two key technologies of this strategy include hydrodissection and intra-procedural contrast-enhanced ultrasound (CEUS), which not only can protect critical structures from conducted heating but also can identify the unablated portion in the same treatment session. Therefore, we combined standard RFA procedure with this strategy to treat large TNs and summarized its clinical outcomes and safety. This study aimed to provide a feasible and effective strategy to resolve the shortcomings of single-session RFA for large TNs.

## Methods

### Patients

All patients were selected with the following inclusion criteria: (a) volume of the TN > 20 ml; (b) confirmation of benignancy (Bethesda Class II) with fine-needle aspiration (FNA) cytology; (c) reports of pressure symptoms or cosmetic problems; (d) anxiety about malignancy; (e) serum levels of thyroid hormone thyrotropin, platelets, blood counts, and blood coagulation tests within normal limits; and (f) patient underwent one single-session RFA treatment.

The exclusion criteria were as follows: (a) substernal nodules or nodules that were difficult to monitor during the RFA procedure; (b) nodules showing malignant features (i.e., taller than wide, speculated margin, marked hypoechoic, or microcalcifications) during US; (c) the patient underwent other treatments for the TN within 6 months before the procedure; and (d) pregnancy.

There were 21 patients with 21 nodules meeting the inclusion criteria from September 2018 to November 2019. This study population consisted of 18 females and 3 males, aged 27–68-years (mean age, 46.1-years).

### Pre-treatment Assessment

Before the procedure, conventional US, US-guided FNA, CEUS, and the laboratory and clinical results were evaluated. Two radiologists (T.W. and J.R. with thyroid US experience of 10 and 18-years, respectively) performed US, US-guided FNA, and CEUS using a Logiq E9 US machine (GE Medical Systems, Milwaukee, WI, USA) equipped with a ML6-15 liner transducer with a center frequency of 7 MHz (frequency range: 2–8 MHz). The US examination included characterization of the position, size, volume, solid/cystic proportions, echogenicity, localization, internal vascularity, and peripheral flow of each nodule. The nodule volume was calculated using the equation: *V* = π × (*a* × *b* × *c*/6) (*V*: volume; *a, b, c*: three diameters of the nodule).

The machine was equipped with CEUS imaging technology. The installed contrast-specific imaging (CSI) mode was coded phase inversion (CPI) at a low mechanical index (<0.2). The contrast agent used in this study was SonoVue (Bracco, Milan, Italy), which contains sulfur hexa?uoride microbubbles. The agent was injected as an intravenous bolus of 2.4 ml into the antecubital vein, followed by a 5-ml 0.9% saline solution flush. The TNs were observed continuously for 2 min to examine the enhanced status and coagulation zone of the nodule with the hybrid contrast visualization mode.

Laboratory tests included the levels of thyroid-stimulating hormone (TSH), thyrotrophin, free triiodothyronine (FT3), and free thyroxin (FT4); a complete blood count; and a coagulation test (prothrombin time, activated partial thromboplastin time). Additionally, all patients underwent vocal cord function assessment performed by an experienced laryngologist before the ablation procedure.

We categorized symptom and cosmetic scores as defined in a previous consensus statement (10). All patients were asked to grade their neck compression discomfort on a scale ranging from 0 to 10 as a symptom score. A cosmetic score was obtained using the following scale: (1) no palpable mass; (2) a palpable mass with no cosmetic problem; (3) a cosmetic problem on swallowing only; (4) readily visible TN.

### Ablation Procedure

**Figure 1:**
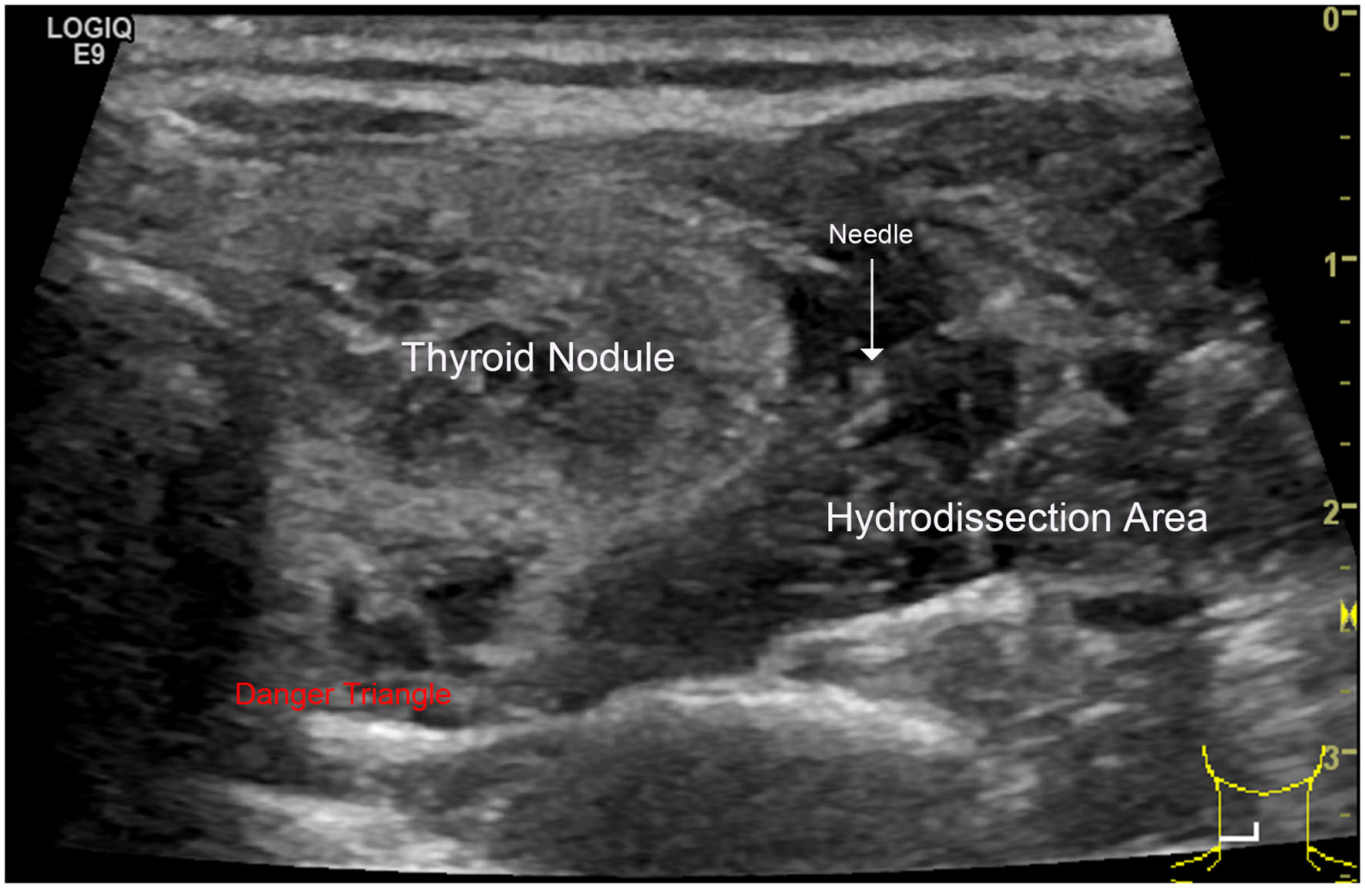
Hydrodissection technique: this technique consists of a pressurized injection of 5% glucose between the danger triangle and the thyroid to reduce possible nerve injury caused by RFA hyperthermia.

All RFA procedures were performed by one radiologist (J.R.) with 3-years of experience in an RFA outpatient clinic. We used an RF generator (Viva RF System®, Starmed, Gyeonggi-si, South Korea) and an internally cooled 18-gauge, 70-mm length, 7- or 10-mm active tip electrode (Star RF Electrode®, Starmed, Gyeonggi-si, South Korea). Local anesthesia with 2% lidocaine was applied to the puncture site. Under US guidance, hydrodissection technique was applied: 5% glucose and norepinephrine were mixed and injected into the surrounding thyroid capsule, which provide a safe distance (>3 mm) between needle tip and adjacent critical structures (Figure 1).

During the procedure, we paid special attention to the preservation of surrounding important structures to prevent significant complications. Therefore, two essential techniques, trans-isthmic or lateral cervical approach and moving-shot technique, were applied. The RFA was performed in transverse US view. Ablation was suspended when the index nodule was covered by hyperechoic zones. After that, initial treatment efficacy was evaluated by CEUS 5–10 min after RFA, until the hyperechoic zones disappeared.

**Figure 2:**
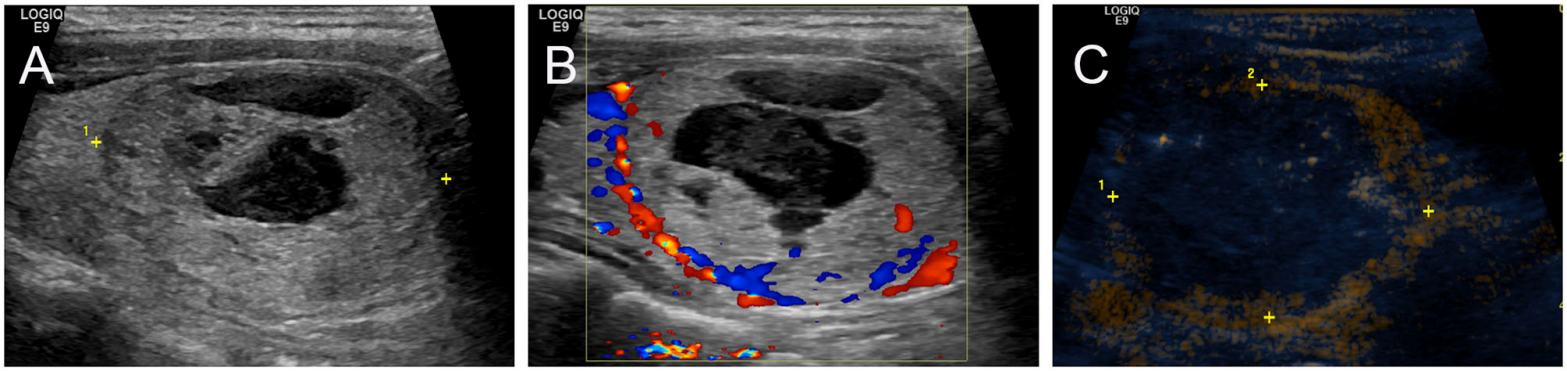
Intraoperative CEUS evaluation: A 38-year-old female had a predominantly solid nodule in the left lobe of the thyroid. The nodule was close to the trachea, carotid artery, and danger triangle area. **(A)** US examination showed that the baseline volume of the nodule was 26.48 ml, and the largest nodule diameter was 4.3 cm. **(B)** CDU showed nodule vascularity was defined as type 1. **(C)** CEUS after initial RFA: the nodule showed non-enhancement during the phase.

When the nodule showed persistence of enhancement after treatment and viable residual tissue on CEUS, CEUS-guided additional ablation was carried out to ablate the enhanced areas, aiming to destroy as much viable residual tissue as possible, while at the same time, treatment safety was the foremost consideration. CEUS was performed again to assess unablated residual tissue after additional RFA (Figure 2).

During treatment, patients' vital signs were continuously monitored (19). The procedure was ended when the nodule presented prevalent devascularization with an unenhanced pattern, the patient exhibited discomfort, or the patient had a high risk of complications that not only interfered with continuation of the procedure but also endangered the patient's life.

### Post-Procedural Follow-Up

Post-procedural follow-up was carried out at 1, 3, and 6 months after treatment. In each follow-up, US examination, CEUS, and thyroid serum tests were performed, symptom score and cosmetic score were evaluated, and VRR of the treated nodule was calculated based on the formula: VRR = [(initial volume – final volume) × 100%]/initial volume. During the follow-up period, any specific complaints or concerns were also recorded.

### Complications and Side Effects

The major complications, minor complications, and side effects are defined by the Society of Interventional Radiology (20). Major complications are defined as events that lead to substantial morbidity and disability, increase the level of care, result in hospital admission, or substantially lengthen the hospital stay. All other complications were considered minor complications. Side effects are defined as untoward consequences that do not require therapy or prescription medications and undesired consequences of the procedure.

### Statistical Analysis

Continuous variables were expressed as means ± standard deviations, and chi-squared tests were used to compare categorical variables. Groups were compared using the Mann–Whitney *U*-test. All statistical analyses were performed using IBM SPSS Statistics 20.0 (Armonk, NY, USA). A *p*-value of 0.05 or less was considered statistically significant.

## Results

### Baseline Characteristics of the Patients and Nodules

The baseline characteristics of the patients and nodules are summarized in Table 1. The mean nodule volume of 27.49 ml corresponds to the mean largest nodule diameter of 4.88 cm. As expected, due to the large nodule volume, TNs were adjacent to at least two important structures, such as the trachea, carotid artery, danger triangle area, thyroid capsule, esophagus, and vagus nerve (Table 1).

**Table 1:** Baseline characteristics of the patients and nodules.

**Variable**	
Number of patients	21
Number of nodules	21
Age (years)	46.05 ± 13.69
Range	27–68
Sex (male/female)	3/18
BMI (kg/m^2^)	22.41 ± 3.65
**Nodule position**	
Left/isthmus/right	12/0/9
Mean nodule volume (ml)	27.49 ± 7.90
Range	20.36–45.72
Mean largest nodule diameter (cm)	4.88 ± 0.77
Range	4–6.9
**Internal nodule component**	
Predominantly solid/predominantly cyst	11/10
Echogenicity	
Hypoechoic/isoechoic/hyperechoic	1/20/0
**Nodules close to dangerous structures**	
Trachea (yes/no)	14/7
Carotid artery (yes/no)	13/8
Danger triangle area (yes/no)	12/9
Esophagus (yes/no)	8/13
Vagus nerve (yes/no)	5/16
**Vascularity**	
0/1/2/3	4/14/3/0
Peripheral flow (yes/no)	11/10
FT3 (pmol/L)	4.37 ± 0.54
FT4 (pmol/L)	13.07 ± 1.95
TSH (mIU/ml)	0.97 ± 0.60
Cosmetic score (=4/ <4)	21/0
Symptom score (≥4/ <4)	4/17

*BMI, body mass index; FT3, free triiodothyronine; FT4, free thyroxin; TSH, thyroid-stimulating hormone*.

### Nodule Treatment Characteristics

The treatment characteristics of the nodules are presented in Table 2. During the procedure, initial treatment efficacy was evaluated by CEUS. Eleven of the 21 TNs underwent additional ablation in the same session.

**Table 2:** Nodule treatment characteristics.

**Variable**	
Mean total energy deposition (kcal)	6.28 ± 3.39
Mean generator time (min)	13.88 ± 7.04
Mean ablation time (min)	61.48 ± 20.31
Additional ablation during the procedure (yes/no)	11/10

### VRR

**Figure 3:**
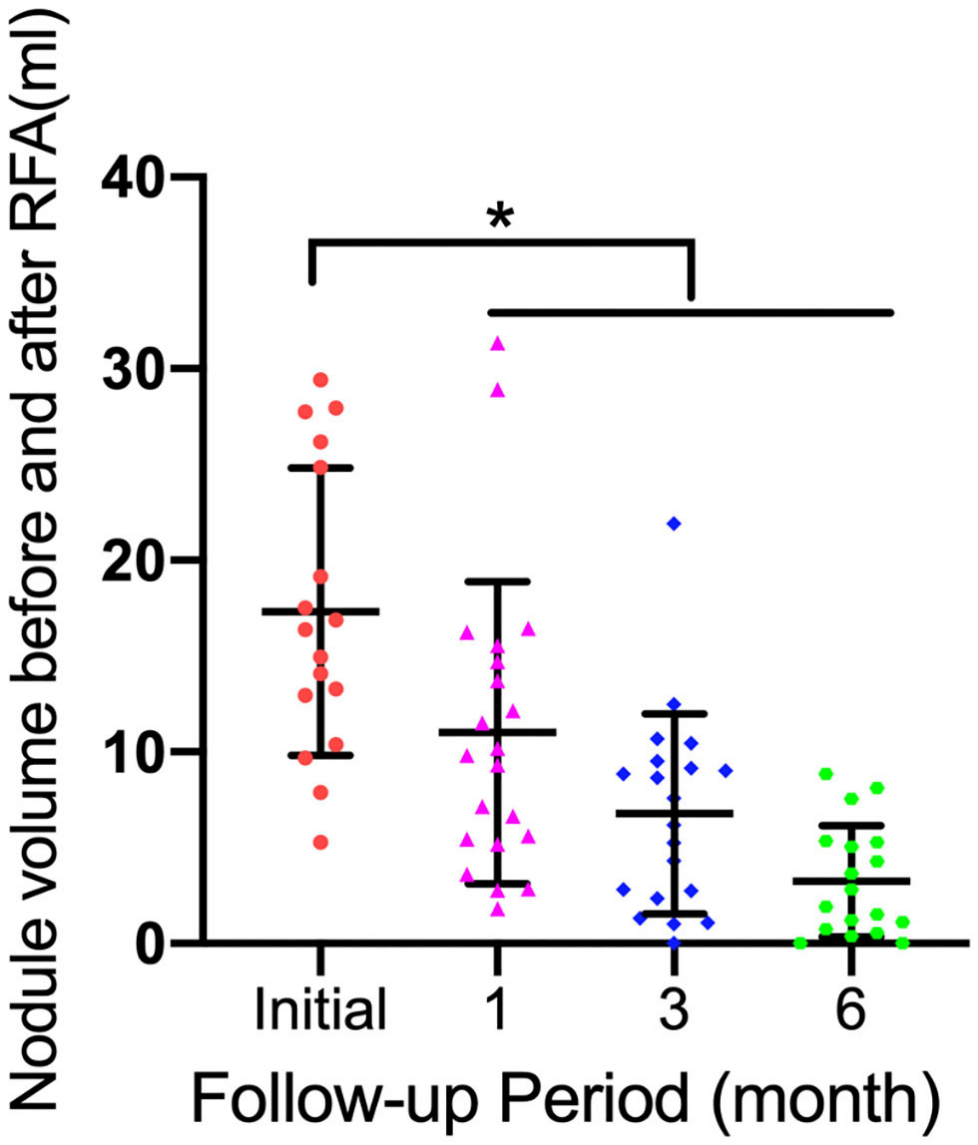
Graphs show mean thyroid nodule volume change during follow-up (FU) and individual nodule volumes. Error bars = standard deviation; *p < 0.05.

The changes in TN volume at each follow-up are summarized in Figure 3. The mean VRRs of all nodules were 60.86 ± 23.25%, 74.71 ± 16.57%, and 83.41 ± 13.96% at the 1, 3, and 6 month follow-ups, respectively (*p* < 0.05) (Figure 3).

### Cosmetic Score and Symptom Score

**Figure 4:**
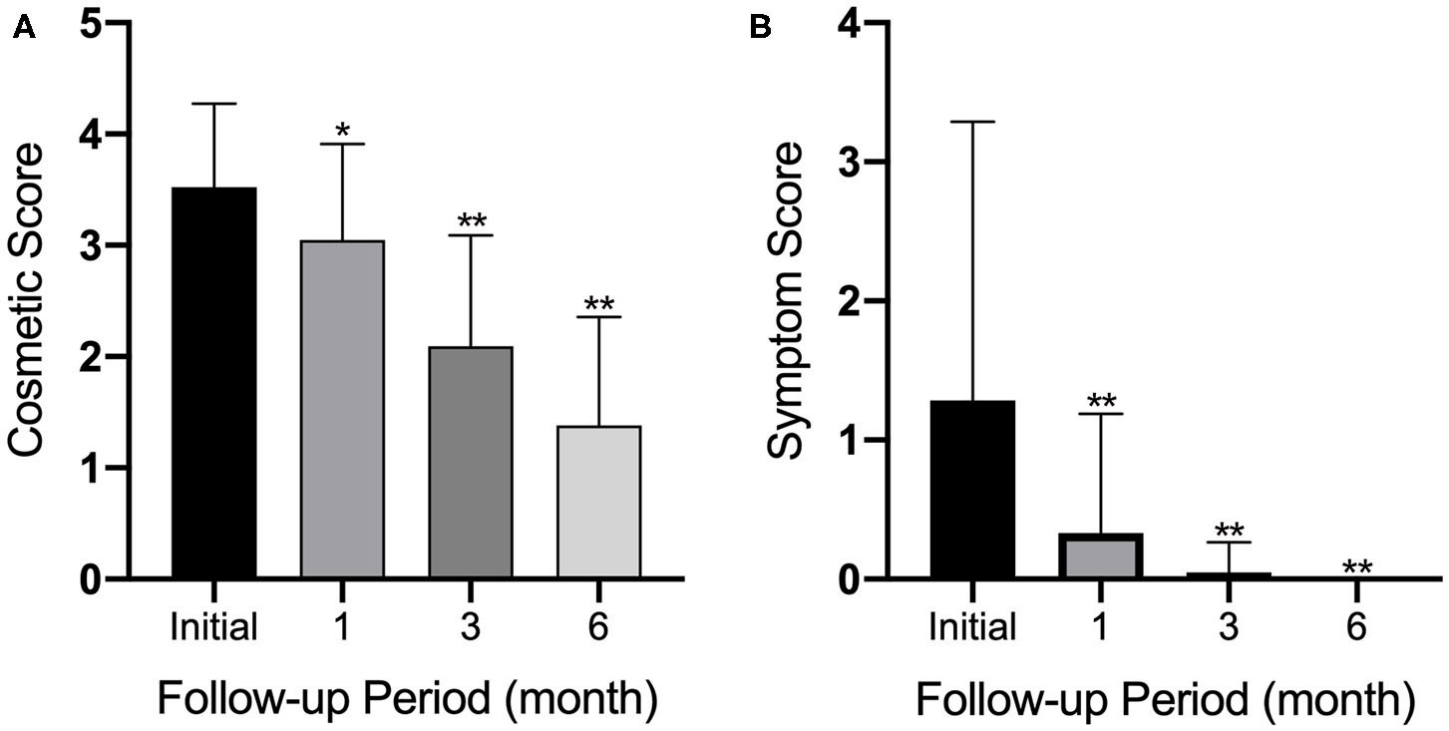
Graphs show **(A)** cosmetic and **(B)** symptom scores at enrollment and at 1, 3, and 6 months after initial RF ablation. Error bars = standard deviation; *p < 0.05; **p < 0.01.

Before RFA, all patients had a readily visible TN. At the 6 month follow-up, this percentage decreased to 5% (*p* < 0.01). Patients with a symptom score of ≥1 (47.6%) at baseline also decreased to 0% at the 6 month follow-up (*p* < 0.01). Patient percentages for cosmetic and symptom scores are provided in Figure 4.

### Complications and Side Effects

Complications and side effects are summarized in Table 3. The overall complication rate was 9.5% (2/21). No patient experienced a life-threatening or delayed complication during follow-up.

**Table 3:** Complications and side effects after RFA.

**List of complications**	**Number of complications**
**Major complication (*****n*** **=** **0)**	
None	0
**Minor complication (*****n*** **=** **2)**	
Voice change (<1 month)	2
**Side effects (*****n*** **=** **5)**	
Vomiting/nausea	1
Pain	4

### Representative Typical Cases

**Figure 5:**
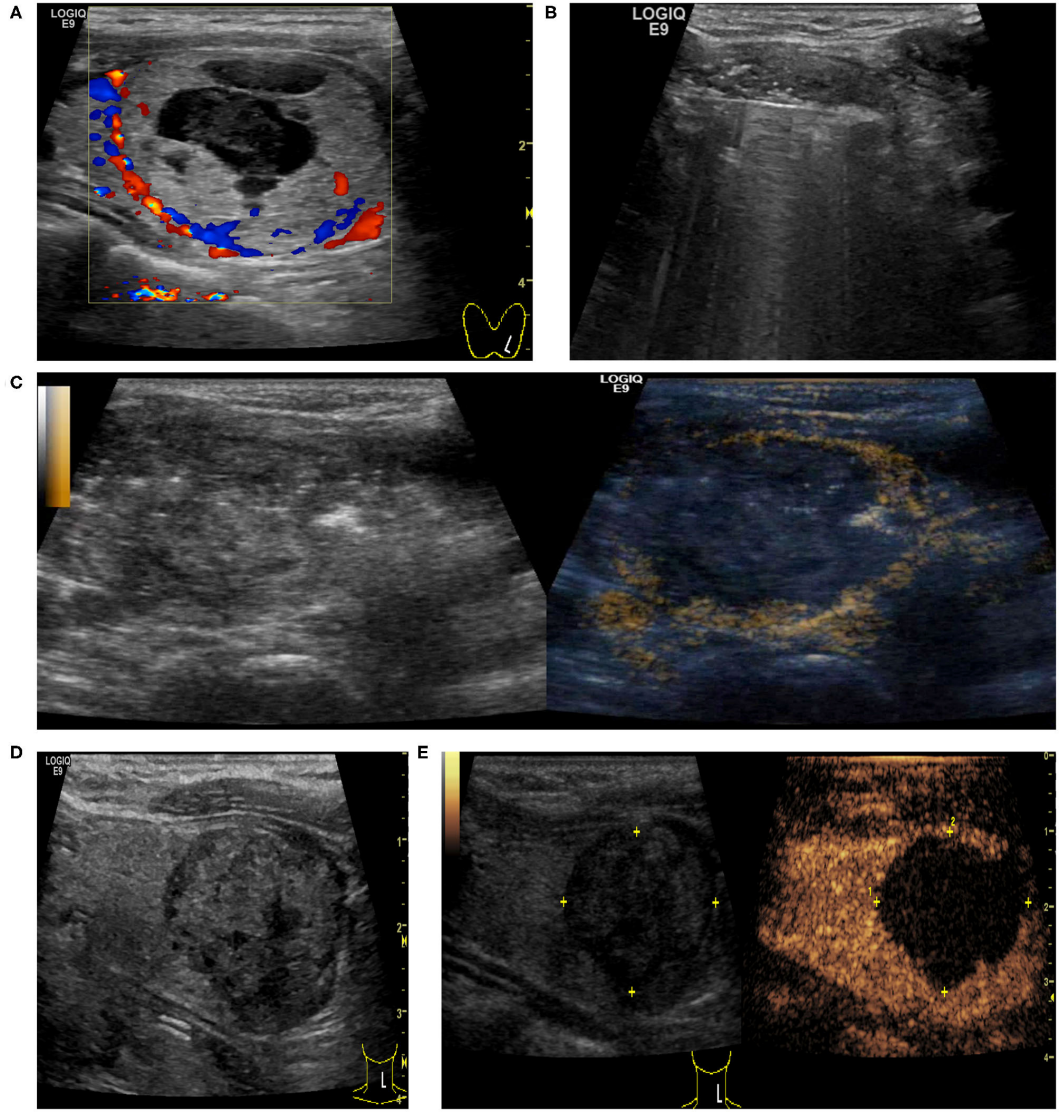
A 38-year-old female had a predominantly solid nodule in the left lobe of the thyroid with a cosmetic score of 4. **(A)** US examination showed that the baseline volume of the nodule was 26.48 ml with the largest nodule diameter of 4.3 cm, and CDU showed nodule vascularity was defined as type 1. **(B)** Ultrasonically guided RFA of thyroid nodule was performed. **(C)** CEUS after initial RFA: some tissue showed enhancement within the nodule, indicating residual tissue. CEUS-guided additional ablation was carried out to ablate the enhanced areas. **(D,E)** At 6 months after ablation, US examination showed that the volume of the nodule decreased to 8.84 ml (the volume reduction rate was 66.6%) and there was no enhancement within the nodule on CEUS.

A 38-year-old woman who had a 43-mm-sized predominantly solid nodule (75% solid component) complained of a foreign body sensation and neck appearance. The nodule in the left lobe of the thyroid was in contact with the lateral wall of the trachea. This patient underwent the procedure with a maximum output of 55 W. After the initial procedure, intra-operative CEUS indicated the presence of residual nodule components. We then performed an additional ablation treatment to achieve complete ablation in a single session (Figure 5).

**Figure 6:**
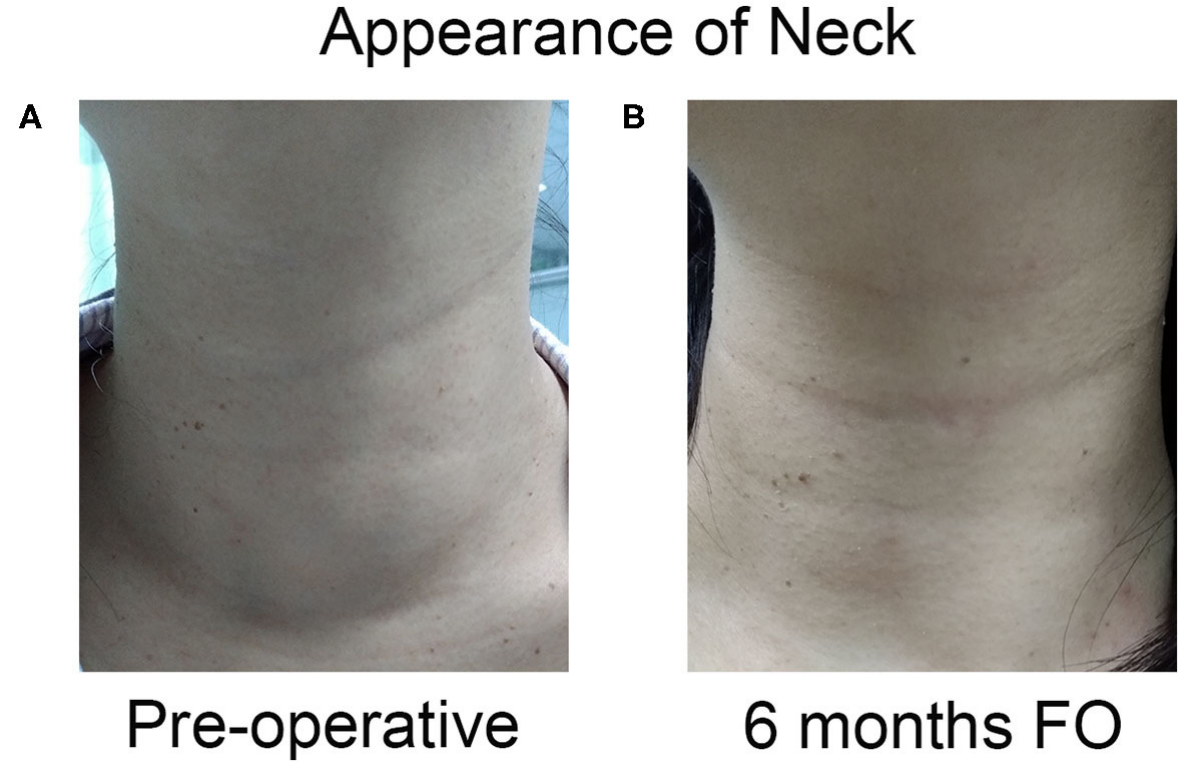
The appearance of neck complaint resolved completely after one session of US-guided RFA. **(A)** The appearance of the neck had a cosmetic score of 4 pre-operation. **(B)** The patient had a good cosmetic score at 6 months after ablation.

Finally, the initial foreign body sensation dissolved completely after 6 months of follow-up (Figure 6).

## Discussion

Recently, lots of minimally invasive treatment modalities, such as ethanol ablation (EA), percutaneous laser ablation (PLA), microwave ablation, and RFA have proven their efficacy and safety as viable TN treatment alternatives to thyroidectomy (21–23). EA is the first-line choice for treating benign cystic nodules (4) but is less effective for solid ones (18). Compared with laser ablation, RFA has shown a superior effect in reducing benign solid TN volume (24) and has a low rate of side effects (25). Since most of the 21 nodules enrolled in this study were solid nodules, RFA was the better option according to some research and guidelines (9, 10).

Despite the fact that these minimally invasive procedures are effective and safe methods of nodule treatment, large TNs (initial volume > 20 ml) consistently influence treatment efficacy and require additional ablation. Del Prete et al. (26) found that EA can be a very effective treatment for thyroid cystic nodules with a 10-year follow-up. However, for TNs with a volume >40 ml, a limited number of sessions (2.7 ± 0.75 sessions) should be performed in order to treat large TNs completely. Cesareo et al. (27) reported that the large nodule subgroup accepting PLA treatment can reach a 60% volume reduction that could be satisfactory, but they still cannot exclude that a second procedure may be necessary considering the large size of these nodules. Huh et al. (16) showed that single-session RFA is effective on most TNs, but for the subgroup with a mean initial volume > 20 ml, additional ablation cannot be avoided if clinical symptoms are to be resolved. Frequently, this subgroup required more than one additional ablation session; in fact, some retrospective studies with long-term follow-up have found that most larger TNs underwent repeated RFA sessions, with some cases requiring as many as seven sessions (17).

Repeat ablation can cause many clinical problems and also has non-medical influence. First, the primary ablation can induce adhesions around the residual tissue, which may cause failure of local anesthesia to prevent pain as well as critical nerve injury caused by conducted heating. Second, the tissue around the residual can become so hard that it inhibits the smooth insertion of the electrode and increases the difficulty of the procedure. Third, the tissue next to the residual can become fibrotic and calcified, a sign of malignancy. These signs may confuse the decision-maker and give rise to some unsatisfactory and unwanted medical disputes, especially in China.

For large TNs, there are two factors associated with the single-session ablation strategy to avoid re-ablation. Safety is the premise of this strategy. The thyroid is a superficial and small organ located in the central neck that is adjacent to several critical structures, such as the common carotid artery, recurrent laryngeal nerve, and vagus nerve. Therefore, despite the nodule location, a large TN will inevitably be close to these vital vessel or nerves (28), and more importantly, a large TN could alter the vagus nerve location, making it closer to the nodule (29). Hereby, we introduce US-guided large volume hydrodissection as a means of protecting the critical structures during our procedure. Hydrodissection has been applied widely in various procedures and surgeries to protect the peripheral nerve (30, 31), and a study has proven the effectiveness of large volume hydrodissection strategy for orthopedics surgery (32). Our research applied continuous D5W (5% dextrose in water) injection to surround the large nodule during the procedure. We found that there were no major complications from injecting as much as 200 ml in the space of the neck, which indicates that large volume hydrodissection can be a feasible approach to protect vital nerves during RFA of large TNs.

Furthermore, incomplete ablation is a common problem for large TNs. The “moving shot” principle is recommended by KSTR guidelines (10) and is more feasible and powerful than fixed electrode techniques (33). Consequently, when multiple ablation units overlap the whole nodule, a complete ablation has occurred. However, it is difficult to avoid leaving a small margin of residual in large TNs monitored only by conventional US surveillance, because the anterior ablation unit with tiny bubble would interfere with the residual definitely. Undoubtedly, the residual will cause regrowth. Lim et al. (17) reported that the overall regrowth rate was 5.6% (7/126) with regrowth defined as a >50% increase in nodule volume compared with the previous follow-up volume. That often means some extent of treatment failure and unsolved clinical problems.

Complete ablation of the periphery of the nodule is the most important factor to prevent marginal regrowth and avoid re-ablation. Therefore, we used intra-procedural CEUS to evaluate the initial treatment response, which facilitated quick decision-making for whether to perform additional ablation in the same session or to finish the procedure and proceed to follow-up. Recently, Zhao et al. (34) reported that some TN factors, including proximity to danger triangle area, proximity to carotid artery, and peripheral blood flow on color-Doppler US, can cause an incomplete ablation; intra-procedural CEUS can be used to avoid this incomplete ablation. Another similar study showed that CEUS could increase the single-session complete ablation rate of US-guided PLA on benign TNs (35). In our research, we found that intra-procedural CEUS can increase the detecting rate of marginal residual after the initial RFA (11/21, 52.4%); consequently, the VRR in this study of large TNs was 60.86 ± 23.25%, 74.71 ± 16.57%, and 83.41 ± 13.96% at 1, 6, and 12 month follow-ups, respectively. To our knowledge, this is a higher VRR for large TN ablation, and more importantly, no major complications occurred in this study. Recently, both Xiaoyin et al. (36) and Cui et al. (19) proved that the strategy of hydrodissection combined with intra-procedural CEUS is safe and effective for relatively large BTNs (diameter > 2 cm). Though the nodules are smaller than our research, it still supports our perspective in some extent.

There were several limitations to the current study. First, this is a retrospective study that may cause some selection bias. Additional prospective studies should be performed in the near future. Next, the case numbers enrolled in this study are relatively small, indicating a need for further research with large case series. In addition, the operator's experience can be a very important factor for the success of this strategy on large TNs, and thus, the operator's level of experience should also be evaluated carefully. Finally, this study's relatively short follow-up period may not be sufficient to assess the real efficacy of this strategy.

## Conclusion

The strategy for single-session complete ablation of large TNs combining RFA with large volume hydrodissection and intra-procedure CEUS, as well as an additional RFA, should be performed in one session. Therefore, large TNs treated with this strategy could achieve a better outcome in terms of VRR compared with the regular method.

## Data Availability Statement

The raw data supporting the conclusions of this article will be made available by the authors, without undue reservation.

## Ethics Statement

The studies involving human participants were reviewed and approved by The Research Ethics Committee of the Third Affiliated Hospital of Sun Yat-sen University. The patients/participants provided their written informed consent to participate in this study.

## Author Contributions

ZY, TW, BL, and JR conceived and designed the project. BZ, LT, and YL acquired the data. TW, BZ, LT, and YL analyzed and interpreted the data. ZY and TW wrote the manuscript. All authors contributed to the article and approved the submitted version.

## Conflict of Interest

The authors declare that the research was conducted in the absence of any commercial or financial relationships that could be construed as a potential conflict of interest.

## References

[1] Moon WJ Baek JH Jung SL Kim DW Kim EK Kim JY. Ultrasonography and the ultrasound-based management of thyroid nodules: consensus statement and recommendations. Korean J Radiol. (2011) 12:1–14. 10.3348/kjr.2011.12.1.1 21228935 PMC3017873

[2] Tunbridge WM Evered DC Hall R Appleton D Brewis M Clark F. The spectrum of thyroid disease in a community: the Whickham survey. Clin Endocrinol. (1977) 7:481–93. 10.1111/j.1365-2265.1977.tb01340.x 598014

[3] Ha EJ Baek JH Lee JH. The efficacy and complications of radiofrequency ablation of thyroid nodules. Curr Opin Endocrinol Diabetes Obes. (2011) 18:310–14. 10.1097/MED.0b013e32834a9168 21841482

[4] Shin JH Baek JH Ha EJ Lee JH. Radiofrequency ablation of thyroid nodules: basic principles and clinical application. Int J Endocrinol. (2012) 2012:919650. 10.1155/2012/919650 23133449 PMC3485526

[5] Jeannon JP Orabi AA Bruch GA Abdalsalam HA Simo R. Diagnosis of recurrent laryngeal nerve palsy after thyroidectomy: a systematic review. Int J Clin Pract. (2009) 63:624–629. 10.1111/j.1742-1241.2008.01875.x 19335706

[6] Gharib H Papini E Paschke R Duick DS Valcavi R Hegedüs L American Association of Clinical Endocrinologists, Associazione Medici Endocrinologi, and European Thyroid Association medical guidelines for clinical practice for the diagnosis and management of thyroid nodules: Executive Summary of recommendations. J Endocrinol Invest. (2010) 33:287–91. 10.1007/BF03346587 20479572

[7] Jeong WK Baek JH Rhim H Kim YS Kwak MS Jeong HJ. Radiofrequency ablation of benign thyroid nodules: safety and imaging follow-up in 236 patients. Eur Radiol. (2008) 18:1244–50. 10.1007/s00330-008-0880-6 18286289

[8] Baek JH Kim YS Lee D Huh JY Lee JH. Benign predominantly solid thyroid nodules: prospective study of efficacy of sonographically guided radiofrequency ablation versus control condition. Am J Roentgenol. (2010) 194:1137–42. 10.2214/AJR.09.3372 20308523

[9] Gharib H Papini E Garber JR Duick DS Harrell RM Hegedüs L. American Association of Clinical Endocrinologists, American College of Endocrinology, and Associazione Medici Endocrinologi medical guidelines for clinical practice for the diagnosis and management of thyroid nodules−2016 update. Endocr Pract. (2016) 22:622–39. 10.4158/EP161208.GL 27167915

[10] Na DG Lee JH Jung SL Kim JH Sung JY Shin JH. Radiofrequency ablation of benign thyroid nodules and recurrent thyroid cancers: consensus statement and recommendations. Korean J Radiol. (2012) 13:117–25. 10.3348/kjr.2012.13.2.117 22438678 PMC3303894

[11] Deandrea M Limone P Basso E Mormile A Ragazzoni F Gamarra E US-guided percutaneous radiofrequency thermal ablation for the treatment of solid benign hyperfunctioning or compressive thyroid nodules. Ultrasound Med Biol. (2008) 34:784–91. 10.1016/j.ultrasmedbio.2007.10.018 18207307

[12] Zhu Y Zhang M Jin Z Tian X Zhang Y Xie F. Solid benign thyroid nodules (>10 ml): a retrospective study on the efficacy and safety of sonographically guided ethanol ablation combined with radiofrequency ablation. Int J Hyperthermia. (2020) 37:157–67. 10.1080/02656736.2020.1717647 32024398

[13] Spiezia S Garberoglio R Milone F Ramundo V Caiazzo C Assanti AP. Thyroid nodules and related symptoms are stably controlled two years after radiofrequency thermal ablation. Thyroid. (2009) 19:219–25. 10.1089/thy.2008.0202 19265492

[14] Baek JH Moon WJ Kim YS Lee JH Lee D. Radiofrequency ablation for the treatment of autonomously functioning thyroid nodules. World J Surg. (2009) 33:1971–7. 10.1007/s00268-009-0130-3 19575141

[15] Cesareo R Naciu AM Iozzino M Pasqualini V Simeoni C Casini A. Nodule size as predictive factor of efficacy of radiofrequency ablation in treating autonomously functioning thyroid nodules. Int J Hyperthermia. (2018) 34:617–23. 10.1080/02656736.2018.1430868 29357717

[16] Huh JY Baek JH Choi H Kim JK Lee JH. Symptomatic benign thyroid nodules: efficacy of additional radiofrequency ablation treatment session–prospective randomized study. Radiology. (2012) 263:909–16. 10.1148/radiol.12111300 22438360

[17] Lim HK Lee JH Ha EJ Sung JY Kim JK Baek JH. Radiofrequency ablation of benign non-functioning thyroid nodules: 4-year follow-up results for 111 patients. Eur Radiol. (2013) 23:1044–9. 10.1007/s00330-012-2671-3 23096937

[18] Lee JH Kim YS Lee D Choi H Yoo H Baek JH. Radiofrequency ablation (RFA) of benign thyroid nodules in patients with incompletely resolved clinical problems after ethanol ablation (EA). World J Surg. (2010) 34:1488–93. 10.1007/s00268-010-0565-6 20376445

[19] Cui D Ding M Tang X Chi J Shi Y Wang T. Efficacy and safety of a combination of hydrodissection and radiofrequency ablation therapy for benign thyroid nodules larger than 2 cm: a retrospective study. J Cancer Res Ther. (2019) 15:386–93. 10.4103/jcrt.JCRT_419_18 30964116

[20] Kim JH Baek JH Lim HK Ahn HS Baek SM Choi YJ. 2017 thyroid radiofrequency ablation guideline: Korean Society of Thyroid Radiology. Korean J Radiol. (2018) 19:632–55. 10.3348/kjr.2018.19.4.632 29962870 PMC6005940

[21] Kim YJ Baek JH Ha EJ Lim HK Lee JH Sung JY. Cystic versus predominantly cystic thyroid nodules: efficacy of ethanol ablation and analysis of related factors. Eur Radiol. (2012) 22:1573–8. 10.1007/s00330-012-2406-5 22437920

[22] Hegedüs L. Therapy: a new nonsurgical therapy option for benign thyroid nodules? Nat Rev Endocrinol. (2009) 5:476–8. 10.1038/nrendo.2009.152 19690558

[23] Feng B Liang P Cheng Z Yu X Yu J Han Z. Ultrasound-guided percutaneous microwave ablation of benign thyroid nodules: experimental and clinical studies. Eur J Endocrinol. (2012) 166:1031–7. 10.1530/EJE-11-0966 22447813

[24] Cesareo R Pacella CM Pasqualini V Campagna G Iozzino M Gallo A. Laser ablation versus radiofrequency ablation for benign non-functioning thyroid nodules: six-month results of a randomized, parallel, open-label, trial (LARA Trial). Thyroid. (2020) 30:847–56. 10.1089/thy.2019.0660 32056501

[25] Mauri G Gennaro N Lee MK Baek JH. Laser and radiofrequency ablations for benign and malignant thyroid tumors. Int J Hyperthermia. (2019) 36:13–20. 10.1080/02656736.2019.1622795 31537159

[26] Del Prete S Caraglia M Russo D Vitale G Giuberti G Marra M. Percutaneous ethanol injection efficacy in the treatment of large symptomatic thyroid cystic nodules: ten-year follow-up of a large series. Thyroid. (2002) 12:815–21. 10.1089/105072502760339398 12481948

[27] Cesareo R Palermo A Pasqualini V Cianni R Gaspa G Manfrini S. Radiofrequency ablation for the management of thyroid nodules: a critical appraisal of the literature. Clin Endocrinol. (2017) 8:639–48. 10.1111/cen.13422 28718950

[28] Baek JH Lee JH Sung JY Bae JI Kim KT Sim JS. Complications encountered in the treatment of benign thyroid nodules with US-guided radiofrequency ablation: a multicenter study. Radiology. (2012) 262:335–42. 10.1148/radiol.11110416 21998044

[29] Ha EJ Baek JH Lee JH. Ultrasonography-based thyroidal and perithyroidal anatomy and its clinical significance. Korean J Radiol. (2015) 16:749–66. 10.3348/kjr.2015.16.4.749 26175574 PMC4499539

[30] Park JK Jeong SY Lee JH Lim GC Chang JW Park JK. Variations in the course of the cervical vagus nerve on thyroid ultrasonography. Am J Neuroradiol. (2011) 32:1178–81. 10.3174/ajnr.A2476 21757523 PMC7966036

[31] Dufour E Donat N Jaziri S Kurdi O Couturier C Dreyfus JF. Ultrasound-guided perineural circumferential median nerve block with and without prior dextrose 5% hydrodissection: a prospective randomized double-blinded noninferiority trial. Anesth Analg. (2012) 115:728–33. 10.1213/ANE.0b013e31825fa37d 22745114

[32] Lee JY Park Y Park KD Lee JK Lim OK. Effectiveness of ultrasound-guided carpal tunnel injection using in-plane ulnar approach: a prospective, randomized, single-blinded study. Medicine. (2014) 93:e350. 10.1097/MD.0000000000000350 25546691 PMC4602597

[33] Ha EJ Baek JH Lee JH. Moving-shot versus fixed electrode techniques for radiofrequency ablation: comparison in an *ex-vivo* bovine liver tissue model. Korean J Radiol. (2014) 15:836–43. 10.3348/kjr.2014.15.6.836 25469097 PMC4248641

[34] Zhao CK Xu HX Lu F Sun LP He YP Guo LH. Factors associated with initial incomplete ablation for benign thyroid nodules after radiofrequency ablation: First results of CEUS evaluation. Clin Hemorheol Microcirc. (2017) 65:393–405. 10.3233/CH-16208 27983547

[35] Ma S Zhou P Wu X Tian S Zhao Y. Detection of the single-session complete ablation rate by contrast-enhanced ultrasound during ultrasound-guided laser ablation for benign thyroid nodules: a prospective study. Biomed Res Int. (2016) 2016:9565364. 10.1155/2016/9565364 27999819 PMC5141549

[36] Xiaoyin T Ping L Dan C Min D Jiachang C Tao W. Risk assessment and hydrodissection technique for radiofrequency ablation of thyroid benign nodules. J Cancer. (2018) 9:3058–66. 10.7150/jca.26060 30210628 PMC6134818

